# Evaluation of the Effectiveness of Surgical Interventions Versus Non-surgical Ones When Used in Conjunction With Fixed Appliances to Accelerate Orthodontic Tooth Movement: A Systematic Review

**DOI:** 10.7759/cureus.25381

**Published:** 2022-05-27

**Authors:** Doa'a Tahseen Alfailany, Mohammad Y Hajeer, Ahmad S Burhan, Luai Mahaini, Khaldoun Darwich, Ossama Aljabban

**Affiliations:** 1 Department of Orthodontics, Faculty of Dentistry, Damascus University, Damascus, SYR; 2 Department of Oral and Maxillofacial Surgery, Faculty of Dentistry, Damascus University, Damascus, SYR; 3 Department of Restorative Dentistry and Endodontics, Faculty of Dentistry, Damascus University, Damascus, SYR

**Keywords:** full-thickness mucoperiosteal flap, mops, piezocision, corticotomy, lllt, prostaglandins, accelerated tooth movement, orthodontic, non-surgical, surgical

## Abstract

The objectives of this review were to evaluate the currently available evidence regarding the effectiveness of surgical versus non-surgical acceleration methods and the side effects associated with these methods. Nine databases were searched: the Cochrane Central Register of Controlled Trials (CENTRAL), EMBASE^®^, Scopus^®^, PubMed^®^, Web of Science™, Google™ Scholar, Trip, OpenGrey, and PQDT OPEN from pro-Quest^®^. ClinicalTrials.gov and the International Clinical Trials Registry Platform Search Portal (ICTRP) were screened to explore ongoing studies and unpublished literature. Randomized controlled trials (RCTs), as well as controlled clinical trials (CCTs) of patients who received surgical interventions (invasive or minimally invasive techniques) in conjunction with traditional fixed appliances and who were compared to the non-surgical interventions, were included. The Cochrane tool for risk of bias (RoB.2) was used for evaluating RCTs, whereas the ROBINS-I tool was used for the CCTs.

This systematic review included four RCTs and two CCTs (154 patients). The surgical and non-surgical interventions were found to have the same effect on orthodontic tooth movement (OTM) accelerating in four trials. In contrast, the surgical interventions were superior in the other two studies. High heterogeneity among the included studies prevented conducting the quantitative synthesis of the findings. The reported side effects related to the surgical and non-surgical interventions were similar.

A "very low" to "low" evidence indicates that the effectiveness of surgical and non-surgical interventions in the acceleration of orthodontic tooth movement is similar, with no differences in the associated side effects. More high-quality clinical trials to compare the acceleration effectiveness between both modalities in different types of malocclusion is required.

## Introduction and background

The treatment time of any orthodontic intervention is one of the essential factors that patients consider when making decisions [1]. For example, canine retraction following upper premolars extraction with maximum anchorage can demand almost seven months, whereas the biological orthodontic tooth movement (OTM) rate is approximately 1 mm over a month, leading to a total treatment duration of about two years [2,3]. Pain, discomfort, dental caries, gingival recession, and root resorption are among the adverse effects of increasing orthodontic treatment duration [4]. Besides, the aesthetic and social reasons lead many patients to ask to finish their orthodontic treatment faster [5]. Therefore, both orthodontists and patients seek to speed up the tooth movement and shorten the treatment time [6].

The approaches to tooth movement acceleration depend on activating biological tissue response. Based on invasiveness, these approaches can be classified into two groups: conservative (biological, physical, and biomechanical methods) and surgical techniques [7].

The biological methods include using pharmacological substances to increase tooth movement in animal experiments and humans. Many studies have found efficacy for most of these substances, such as cytokines, receptor activator of nuclear factor kappa-Β ligand/receptor activator of nuclear factor kappa-Β (RANKL/RANK) protein, prostaglandins, vitamin D, hormones such as parathyroid hormone (PTH), and osteocalcin, whereas injection of other substances such as relaxin did not show any accelerating efficacy [8].

The physical methods depend on employing device-assisted therapy and involve direct electric currents [9], pulsed electromagnetic field [10], vibration [11], and low-level laser therapy [12], which showed promising results after being verified [8]. The surgical methods are considered the most clinically used and most tested with the possibility of significantly reducing the duration of treatment [13,14]. However, they depend on the "regional acceleratory phenomenon (RAP)," as the occurrence of surgical damage to the alveolar bone may temporarily accelerate the OTM [15]. These surgical interventions involve conventional corticotomy [16,17], interseptal alveolar surgery [18], accelerated osteogenic orthodontics [19], dentoalveolar distraction [13], and periodontal distraction [20], piezocision [14,21], corticision [22], and micro-osteoperforations [23].

Several systematic reviews (SRs) of randomized controlled trials (RCTs) have been published about the effectiveness of both surgical and non-surgical interventions in the acceleration of OTM [24,25]. However, the superiority of the surgical over the non-surgical techniques has not been proven yet. Therefore, this systematic review (SR) intended to answer the following focused review question - which is more effective in accelerating orthodontic tooth movement when using fixed orthodontic appliances: surgical or non-surgical techniques?

## Review

Preliminary search and protocol registration

First of all, to make sure that there are no similar SRs and to check for any relevant articles before writing the final protocol of the SR, a PubMed pilot search was conducted. Later, two potentially competent trials were found and assessed. The registration of this SR protocol at the PROSPERO database was done (ID number: CRD42021274312). This SR was constructed under the Cochrane Handbook for Systematic Reviews of Interventions [26] and the Preferred Reporting Items for Systematic Reviews and Meta-Analyses (PRISMA) guidelines [27,28].

Eligibility criteria

Inclusion Criteria

According to participants, interventions, comparisons, outcomes, and study design (PICOS) framework, male and female healthy patients, regardless of their age, type of malocclusion, or racial group, who underwent fixed orthodontic treatment were included. Surgical interventions (invasive or minimally invasive) adjunct to traditional fixed orthodontic treatment were considered. Patients receiving fixed orthodontic treatment (OT) combined with non-surgical interventions were included. These interventions could include pharmacological methods (local or systemic) and physical methods (laser irradiation, electric currents, pulsed electromagnetic field (PEMF), and vibration).

The primary outcome of this criteria was the rate of tooth movement (RTM) or any synonymous measurement that could inform us about the effectiveness of the surgical and non-surgical intervention. The secondary outcomes include untoward effects like patient-reported outcomes (pain, discomfort, satisfaction, oral-health-related quality of life, difficulties in mastication, and other experiences), complications related to periodontal tissues assessed by periodontal index (PI), gingival index (GI), attachment loss (AT), gingival recession (GR), periodontal depth (PD), anchorage loss and undesired tooth movement (tipping, torquing, rotation) or iatrogenic teeth injuries such as loss of tooth vitality, resorption of roots. Only two study designs were accepted for inclusion - randomized controlled trials (RCTs) and clinical controlled trials (CCTs) written only in the English language and without any restrictions on publication year.

Exclusion Criteria

The following articles were excluded: retrospective studies, non‑English language studies, animal trials, in vitro studies, case reports or case series reports, editorials, reviews and technique description articles, personal opinions, trials without a reported sample, absence of a control group or the presence of a control group of non-treated patients, fewer than 10 patients in the experimental group, and finite element analysis studies.

Search strategy

An electronic search was created within the subsequent databases (in August 2021 with no time limitations and only in the English language): the Cochrane Central Register of Controlled Trials, PubMed®, Scopus®, Web of Science™, EMBASE®, Google™ Scholar, Trip, OpenGrey (to identify the grey literature), and PQDT OPEN from pro-Quest® (to identify dissertations and theses). The reference lists of chosen papers were also screened for any potentially related trials that may the electronic web-based search has not discovered. In the same time frame, manual searching was done in the Angle Orthodontist journal, the American Journal of Orthodontics and Dentofacial Orthopedics™, the European Journal of Orthodontics, and the Orthodontics and Craniofacial Research. ClinicalTrials.gov and World Health Organization International Clinical Trials Registry Platform Search Portal (ICTRP) underwent an electronic check to regain unpublished trials or presently achieved research studies. More details about the electronic search strategy are presented in Table 1.

**Table 1:** Electronic search strategy RANKL: receptor activator of nuclear factor kappa-Β ligand; RANK: receptor activator of nuclear factor kappa-Β

Database	Search strategy
CENTRAL	#1 orthodontic* OR "tooth movement" OR "orthodontic tooth movement” OR "orthodontic treatment" OR "orthodontic therapy" #2 accelerat* OR rapid* OR short* OR speed* OR fast OR velocity OR duration OR rate OR time OR "regional accelerated phenomenon" OR RAP. #3 (surgical( AND (corticotom* OR decorticat* OR alveolar surg* OR piezosurgery OR piezoelectric OR piezo* OR microsurgery OR micro incisions OR micro osteoperforations OR micro perforations OR perforations OR corticision OR lasercision OR corticopuncture OR piezocision OR piezotome OR piezopuncture) #4 (non-surgical( AND (pharmacological OR physical OR device-assisted therapy OR cytokines OR RANKL/RANK protein OR prostaglandins OR vitamin D OR PTH hormone OR osteocalcin OR relaxin OR low level laser therapy OR LLL OR LLLT OR photobiomodulation OR direct electric currents OR pulsed electromagnetic field OR vibration) #5 #2 OR #3 OR #4 #6 #1 AND #5
EMBASE	#1 orthodontic* OR "tooth movement" OR "orthodontic tooth movement” OR "orthodontic treatment" OR "orthodontic therapy" #2 accelerat* OR rapid* OR short* OR speed* OR fast OR velocity OR duration OR rate OR time OR "regional accelerated phenomenon" OR RAP. #3 (surgical( AND (corticotom* OR decorticat* OR alveolar surg* OR piezosurgery OR piezoelectric OR piezo* OR microsurgery OR micro incisions OR micro osteoperforations OR micro perforations OR perforations OR corticision OR lasercision OR corticopuncture OR piezocision OR piezotome OR piezopuncture) #4 (non-surgical( AND (pharmacological OR physical OR device-assisted therapy OR cytokines OR RANKL/RANK protein OR prostaglandins OR vitamin D OR PTH hormone OR osteocalcin OR relaxin OR low level laser therapy OR LLL OR LLLT OR photobiomodulation OR direct electric currents OR pulsed electromagnetic field OR vibration) #5 #2 OR #3 OR #4 #6 #1 AND #5
PubMed	#1 orthodontic* OR "tooth movement" OR "orthodontic tooth movement” OR "orthodontic treatment" OR "orthodontic therapy" #2 accelerat* OR rapid* OR short* OR speed* OR fast OR velocity OR duration OR rate OR time OR "regional accelerated phenomenon" OR RAP. #3 (surgical( AND (corticotom* OR decorticat* OR alveolar surg* OR piezosurgery OR piezoelectric OR piezo* OR microsurgery OR micro incisions OR micro osteoperforations OR micro perforations OR perforations OR corticision OR lasercision OR corticopuncture OR piezocision OR piezotome OR piezopuncture) #4 (non-surgical( AND (pharmacological OR physical OR device-assisted therapy OR cytokines OR RANKL/RANK protein OR prostaglandins OR vitamin D OR PTH hormone OR osteocalcin OR relaxin OR low level laser therapy OR LLL OR LLLT OR photobiomodulation OR direct electric currents OR pulsed electromagnetic field OR vibration) #5 #2 OR #3 OR #4 #6 #1 AND #5
Scopus	#1TITLE-ABS-KEY (orthodontic* OR "tooth movement" OR "orthodontic tooth movement” OR "orthodontic treatment" OR "orthodontic therapy"). #2TITLE-ABS-KEY (accelerat* OR rapid* OR short* OR speed* OR fast OR velocity OR duration OR rate OR time OR "regional accelerated phenomenon" OR RAP). #3 TITLE-ABS-KEY ("surgical") AND TITLE-ABS-KEY (corticotom* OR decorticat* OR alveolar surg* OR piezosurgery OR piezoelectric OR piezo* OR microsurgery OR micro incisions OR micro osteoperforations OR micro perforations OR perforations OR corticision OR lasercision OR corticopuncture OR piezocision OR piezotome OR piezopuncture). #4 TITLE-ABS-KEY ("non-surgical ") AND TITLE-ABS-KEY (pharmacological OR physical OR device-assisted therapy OR cytokines OR RANKL/RANK protein OR prostaglandins OR vitamin D OR PTH hormone OR osteocalcin OR relaxin OR low level laser therapy OR LLL OR LLLT OR photobiomodulation OR direct electric currents OR pulsed electromagnetic field OR vibration). #5 #2 OR #3 OR #4 #6 #1 AND #5
Web of Science	#1TS= (orthodontic OR "tooth movement" OR "orthodontic tooth movement” OR "tooth displacement “OR "orthodontic treatment" OR "orthodontic therapy"). #2TS= (accelerat* OR rapid* OR short* OR speed* OR fast OR velocity OR duration OR rate OR time OR "regional accelerated phenomenon" OR RAP). #3TS= (surgical (AND TS= (corticotom* OR decorticat* OR alveolar surg* OR piezosurgery OR piezoelectric OR piezo* OR microsurgery OR micro incisions OR micro osteoperforations OR micro perforations OR perforations OR corticision OR lasercision OR corticopuncture OR piezocision OR piezotome OR piezopuncture). #4TS= (non-surgical (AND TS= (pharmacological OR physical OR device-assisted therapy OR cytokines OR RANKL/RANK protein OR prostaglandins OR vitamin D OR PTH hormone OR osteocalcin OR relaxin OR low level laser therapy OR LLL OR LLLT OR photobiomodulation OR direct electric currents OR pulsed electromagnetic field OR vibration). #5 #2 OR #3 OR #4 #6 #1 AND #5
Google Scholar	#1 (orthodontic OR "tooth movement" OR "orthodontic tooth movement” OR "tooth displacement “OR "orthodontic treatment" OR "orthodontic therapy") AND (accelerate OR acceleration OR accelerating OR accelerated OR rapid) AND (surgical ) AND (non-surgical) #2 (orthodontic OR "tooth movement" OR "orthodontic tooth movement” OR "tooth displacement “OR "orthodontic treatment" OR "orthodontic therapy") AND (accelerate OR acceleration OR accelerating OR accelerated OR rapid) AND (corticotom* OR decorticat* OR alveolar surg* OR piezosurgery OR piezoelectric OR piezo* OR microsurgery OR micro incisions OR micro osteoperforations OR micro perforations OR perforations OR corticision OR lasercision OR corticopuncture OR piezocision OR piezotome OR piezopuncture) AND (pharmacological OR physical OR device-assisted therapy OR cytokines OR RANKL/RANK protein OR prostaglandins OR vitamin D OR PTH hormone OR osteocalcin OR relaxin OR low level laser therapy OR LLL OR LLLT OR photobiomodulation OR direct electric currents OR pulsed electromagnetic field OR vibration)
Trip	(orthodontic* OR "tooth movement" OR "orthodontic tooth movement” OR "orthodontic treatment" OR "orthodontic therapy") AND (accelerat* OR rapid* OR short* OR speed* OR fast OR velocity OR duration OR rate OR time OR "regional accelerated phenomenon" OR RAP) AND (corticotom* OR decorticat* OR alveolar surg* OR piezosurgery OR piezoelectric OR piezo* OR microsurgery OR micro incisions OR micro osteoperforations OR micro perforations OR perforations OR corticision OR lasercision OR corticopuncture OR piezocision OR piezotome OR piezopuncture) AND (pharmacological OR physical OR device-assisted therapy OR LLLT OR LLL OR low level laser therapy).
OpenGrey	#1 acceleration AND tooth movement #2 orthodontic AND acceleration #3 corticotom* OR decorticat* OR alveolar surg* OR piezosurgery OR piezoelectric OR piezo* OR microsurgery OR micro incisions OR micro osteoperforations OR micro perforations OR perforations OR corticision OR lasercision OR corticopuncture OR piezocision OR piezotome OR piezopuncture. #4 pharmacological OR physical OR device-assisted therapy OR cytokines OR RANKL/RANK protein OR prostaglandins OR vitamin D OR PTH hormone OR osteocalcin OR relaxin OR low level laser therapy OR LLL OR LLLT OR photobiomodulation OR direct electric currents OR pulsed electromagnetic field OR vibration
PQDT OPEN	#1 acceleration AND tooth movement #2 orthodontic AND acceleration
World Health Organization (WHO) International Clinical Trials Registry Platform (ICTRP)	(orthodontic OR “dental movement” OR “tooth movement” OR “orthodontic tooth movement”) AND (accelerat* OR rapid* OR short* OR speed* OR fast)
ClinicalTrials.gov	(orthodontic OR “dental movement” OR “tooth movement” OR “orthodontic tooth movement”) AND (accelerat* OR rapid* OR short* OR speed* OR fast)

Study selection and data extraction

Two reviewers (DTA and MYH) independently estimated the eligibility of the trials, and in case of difference, a third author (LM) was asked to arrive at a decision. The first step included checking the titles and abstracts only. The second step for all studies was the assessment of full-text appearing to be pertinent and filtering for inclusion, or when the title or abstract was unclear to help reach a clear judgment. when papers did not achieve one or more of the inclusion criteria, they were excluded. For more explanation or additional data, corresponding authors were e-mailed. The same authors (DTA and MYH) extracted data independently in the piloted and predefined data extraction tables. When a disagreement between the two main reviewers occurred, a third author (LM) was asked for help to resolve it. The data summary sheet included the following items: general information about the article (author(s)' name, publication year, and study setting); methods (study design, groups being evaluated); participants (number of recruited patients, mean age, and age range, gender); intervention (the type of procedures, location of the procedure, technical aspects of procedures); orthodontic characteristics (malocclusion class, type of orthodontic tooth movement, frequency of orthodontic adjustments, time of follow-up); and outcome measures (primary and secondary outcomes mentioned, methods of measuring, the reported statistically significant difference).

Evaluation of the risk of bias and strength of evidence

Two reviewers (DTA and MYH) assessed the risk of bias using the RoB-2 tool for the retrieved RCTs [29] and the ROBINS-I tool for the CCTs [30]. In case of disagreement, one of the co-authors (ASB) was consulted to reach a solution. We assessed the following domains as at "low," "high risk" or "some concern of bias" for randomized trials: bias arising from the randomization process, bias due to deviations from intended interventions (effect of assignment to intervention; effect of adhering to intervention), bias due to missing outcome data, bias in the measurement of the outcome, bias in the selection of the reported result. The overall risk-of-bias judgment of the chosen studies was assessed as follows: "low risk of bias" if all fields were evaluated as "at low risk of bias"; "some concerns" if at least one domain was assessed as "some concerns," but not to be "at high risk of bias for any domain, high risk of bias: if at least one or more fields were evaluated as at high risk of bias" or there some concerns for multiple domains in a way that substantially lowers confidence in the result. Whereas, for the non-randomized trials, we evaluated the following domains as low, moderate, and serious risk: pre-intervention (bias due to confounding; bias in the selection of participants into the study); at intervention (bias in the classification of interventions); post-intervention (bias due to deviations from intended interventions; bias due to missing data; bias in the measurement of outcomes; bias in the selection of the reported result). The overall risk-of-bias judgment of the chosen studies was assessed as follows: "low risk of bias" if all domains were evaluated as "at low risk of bias"; "moderate risk of bias" if all domains were evaluated as "the low or moderate risk of bias"; "serious risk of bias" if at least one domain assessed as "serious risk of bias" but not at critical risk of bias in any domain, "critical risk of bias" if at least one domain assessed as "critical risk of bias"; "no information" if there is no clear indication that the study is "at serious or critical risk of bias" and there is a lack of information in one or more key domains of bias. The strength of evidence was evaluated according to the Grading of Recommendations Assessment, Development and Evaluation (GRADE) approach, as high, moderate, low, or very low for the outcomes [31].

Results

Study Selection and the Literature Flow

**Figure 1:**
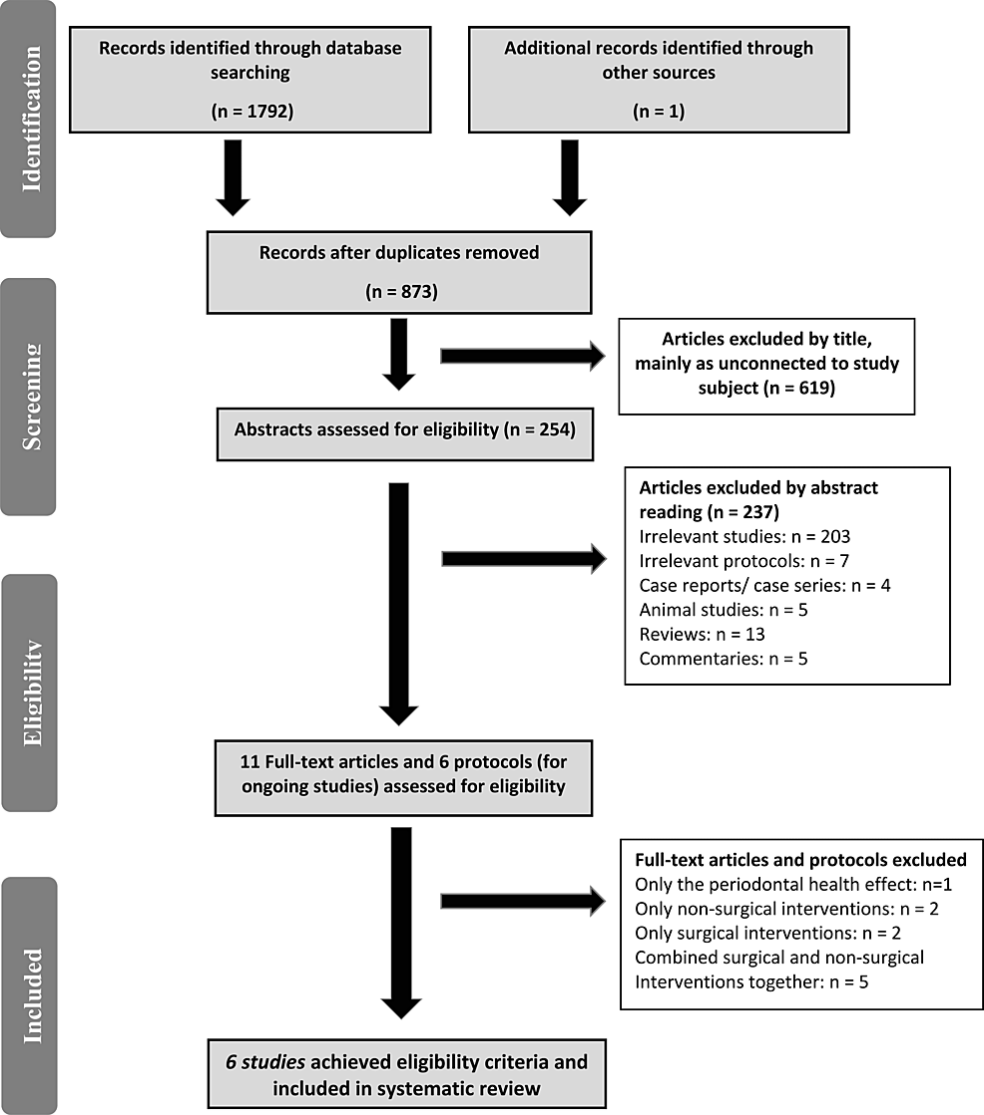
Preferred Reporting Items for Systematic Reviews and Meta-Analyses (PRISMA) flow diagram of the included studies

After the electronic search, 1972 articles were identified, as well as only one more reference was found from other sources. After taking off the duplicates, 873 articles were reviewed. The titles and abstracts were examined for eligibility, and then all studies, which failed to meet the eligibility criteria, were discarded. As a result, 11 potentially relevant documents were checked in depth. Five completed trials and five of the ongoing studies did not match the inclusion criteria. A summary of the excluded articles after full-text assessment with reasons for exclusion is presented in the table in Appendices. Finally, six studies (four RCTs and two CCTs) were included in the SR [23,32-36]. The PRISMA flow diagram is presented in Figure 1.

Characteristics of the Included Studies

The characteristics of the six included trials are given in Table 2 and Table 3 [23,32-36]. Only one protocol trial was found; more information about that ongoing research project is shown in Table 4 and Table 5.

**Table 2:** Characteristics of the included studies: PICOS, follow-up period, and main findings RCT: randomized clinical trial; NAC: non-accelerated control; SMD: split-mouth design; MOPs: micro-osteoperforations; LLLT: low-level laser therapy; CFO: corticotomy-facilitated orthodontics; FTMPF: full-thickness mucoperiosteal flap; Exp: experimental; M: male; F: female; U3: upper canine; ED: energy density; RTM: rate of tooth movement; TTM: time of tooth movement; CTM: cumulative tooth movement; PICOS: participants, interventions, comparisons, outcomes, and study design Compound design: it consists of both parallel and split-mouth

Study/setting	Methods		Participants	Type of malocclusion	Interventions			Outcomes
	Study design	Treatment comparison	Patients (M/F) age (years)		Type and site of intervention/technical aspects of interventions	Application frequency	Follow-up time	Primary and secondary outcomes
Rajasekaran and Nayak, 2014 [35] India	CCT (split-mouth design)	Corticotomy vs. prostaglandin E1	Patients (M/F): 32 (17\ 15) Control: 32 Exp: 32 Age (years): 18-24	Patients who need to extract maxillary 1st premolars and maxillary canine retraction	- Prostaglandin: 0.2 ml of prostaglandin E1 was injected on the buccal side adjacent to the right U3. - Corticotomy: after full-thickness flap, a vertical bony cut adjacent to the distal U3 root on the buccal, and palatal surfaces were done using a surgical bone-cutting bur.	Prostaglandin: once in 2 weeks till completion of canine retraction	Until completion of space closure of the extraction sites	Primary outcome: -RTM (mm/week) -TTM (weeks) Secondary outcomes: -Molar anchorage loss. -The crestal bone height changes. -Root length. -Pain and swelling
Abdelhameed and Refai, 2018 [23] Minya, Egypt	RCT (compound design)	(MOPs\NAC) vs. (LLLT\NAC) vs. (MOPs + LLLT\NAC)	Patients (M/F): 30 (NR\ NR) Control: 30, Exp: 30 Age (years): 15-25	Patients who need to extract maxillary 1st premolars and maxillary canine retraction	- MOPs: 12 MOPs with a depth of 6 mm were applied by mini-screws (Six MOPS were done buccally and six palatally). - LLLT: a soft laser (wavelength: 810 ± 10 nm) was used from buccal and palatal surfaces along the root of the U3.	MOPs: The technique was repeated every two weeks. LLLT: The application of laser was at the beginning of a canine retraction, after three days, one week, two weeks, then every two weeks along three months.	3 months	Primary outcome: -RTM (mm/week)
El-Ashmawi et al., 2018 [33] Cairo, Egypt	RCT (split-mouth design)	Corticotomy vs. LLLT	Patients (M/F): 20 (1\ 19) Control: 20, Exp: 20 Age (years): 16-25	Patients who need to extract maxillary 1st premolars and maxillary canine retraction	- Corticotomy: After an elevated flap, 10 to 15 corticotomy perforations were made around the root of the U3, using surgical bur. - LLLT: InGaAs diode laser (wavelength: 940 ± 10 nm, ED: 29.3 J/cm^2^) was applied on the buccal mucosa at the middle point of the U3 root for 300 seconds.	LLLT: The application of laser was On the day of first premolars extraction, then after 1, 2, 3, weeks, then every 2 weeks until the end of the study.	4 months	Primary outcome: -RTM (mm/week) Secondary outcomes: -Molar anchorage loss. -post-surgical swelling and pain -sensitivity in the maxillary lateral incisor -ulcers
Sedky et al., 2019 [34] Cairo, Egypt	RCT (split-mouth design)	CFO vs. LLLT	Patients (M/F): 20 (8\ 12) Control: 20, Exp: 20 Age (years): 18-29	patients who need to extract maxillary 1st premolars and maxillary canine retraction	- CFO: After elevated a full-thickness flap, corticotomy perforations were made extending from the lateral incisor to the first premolar area using a round bur with a depth of approximated the width of the buccal cortical bone. - LLLT: InGaAs diode laser (wavelength: 940 nm, ED: 3.937 J/cm^2^) was applied on the buccal mucosa at the middle point of the U3 root for 25 seconds.	LLLT: The application of laser was at the beginning of a canine retraction, then the irradiation was repeated on days 3, 8, and 15.	Two weeks	Primary outcome: -sRANKL concentration (pg\ml).
Abdarazik et al., 2020 [32] Cairo, Egypt	RCT (compound design)	(FTMPF\ NAC) vs. (LLLT\ NAC)	Patients (M/F): 32 (0\ 32) Control: 16, Exp: 16 Age (years): 15-20	patients who need to extract maxillary 1st premolars and maxillary canine retraction	- FTMPF: A full-thickness mucoperiosteal flap (FTMPF) was done from the distal surface of the maxillary second premolar to the mesial side of the U3 with an elevation of palatal mucosa. - LLLT: In-Ga-As diode laser (wavelength: 940 nm, ED: 4.2 J/cm^2^) was applied for 60 seconds.	LLLT: The application of laser was done after extraction on days 3, 7, 14, 28, 42,56.	Until completion of canine retraction.	Primary outcome: -CTM (mm) -TTM (weeks) Secondary outcomes: -Molar anchorage loss. - Periodontal probing depth.
Türker et al., 2020 [36] Kayseri, Turkey	CCT (split-mouth design)	Piezocision vs. LLLT	Patients (M/F): 20 (5\ 15) Control: 20, Exp: 20 Mean age (years): 16.35± 1.14	patients who need to extract maxillary 1st premolars and maxillary canine retraction	- Piezocision: Two vertical interproximal incisions were made (3-mm depth and 3-5-mm length) on the distobuccal and mesiobuccal sides of the right U3. LLLT: Diode laser (wavelength: 940 nm, ED: 5 J/cm^2^) was applied at 8 sides (4 buccal and 4 palatal) around left U3 for 80 seconds.	LLLT: The application of laser was done on day 0 and days 3, 7, 14, 21, and 28 after the start of canine retraction in the first month.	3 months	Primary outcome: -RTM (mm/week) Secondary outcomes: -Canine and 1^st^ molar angulation.

**Table 3:** Additional characteristics of the included studies (appliance and anchorage used, orthodontic adjustments, statistical outcomes, and methods of primary outcome measurements) TADs: temporary anchorage devices; RTM: rate of tooth movement; TTM: time of tooth movement; CTM: cumulative tooth movement; EXP: experimental; NR: not reported; U3: upper canines; U6: upper first molar; SS: stainless steel; NiTi: nickel titanium; MOPs: micro-osteoperforations; LLLT: low-level laser therapy; CFO: corticotomy-facilitated orthodontics; FTMPF: full-thickness mucoperiosteal flap

Authors (year, country)	Appliance characteristics	Anchorage used	Orthodontic adjustments	Statistical significance of reported outcomes		Methods of primary outcome measurements
				Primary outcomes	Secondary outcomes	
Rajasekaran and Nayak, 2014 [35] India	- MBT prescription brackets+ 0.017 × 0.025-inch SS+ NiTi closed-coil springs (100 g) between the hooks of U6 band and the U3 bracket for retraction U3.	A tip backbend.	Every week	RTM (mm/week): p-value = 0.003 Corticotomy: 0.40 ± 0.04, prostaglandin: 0.36 ± 0.05 TTM (weeks): (p-value = NR) Corticotomy: 13 prostaglandin: 15	Molar anchorage los: (p-value = 0.67) Using cast, no statistically significant difference between the two groups. The crestal bone height changes and root length: (p-value =0.08) Using IOPAs, no statistically significant difference between the two groups.	Study models using electric digital calipers
Abdelhameed and Refai, 2018 [23] Minya, Egypt	- MBT prescription brackets+ NiTi closed-coil springs (150g) between the hook of U3 and mini-screw for retraction U3.	TADs between 5 and 6	Every two weeks	RTM (mm/week): 2nd, 4th, 6th week: (MOPs) p-value = 0.000 /8th, 10th, 12th week: (MOPs) p-value = 0.001 2nd, 4th, 6th, 8th, 10th, 12th week: (LLLT) p-value = 0.001		Direct intra-oral measurements using a digital intra-oral caliper
El-Ashmawi et al., 2018 [33] Cairo, Egypt	- MBT prescription brackets+ 0.016 × 0.022-inch SS+ NiTi closed-coil springs (150g) between the hooks of U6 band and the U3 bracket for retraction U3.	TADs between 5 and 6	Every two weeks (except 1 week after the surgery to stabilize the surgical flap)	RTM (mm/week): p-value >0.05 Corticotomy: 4.32±1.29, LLLT: 4.55± 1.7	Molar anchorage loss: (p-value = 0.45) Using cast, no statistically significant difference between the two groups.	-Measurements were done using 2D-scanned models and digital calipers. - CBCT to assess 3D movements of the U3.
Sedky et al., 2019 [34] Cairo, Egypt	- Roth prescription brackets+ 0.016 × 0.022-inch SS+ NiTi closed-coil springs (150 g) between the hook of U3 and mini-screw for retraction U3.	TADs between 5 and 6	day before intervention, 3rd and 15th day after intervention	sRANKL concentration (pg\ml): 1 day before intervention: p-value (NR) (CFO): 3.8 ± 1.4, (LLLT): 3.8 ± 1.4 3rd day after intervention: p-value =0.685 (CFO): 10.2 ± 1.2, (LLLT): 10.0 ± 1.9 15th day after intervention: p-value =0.400 (CFO): 8.9 ± 1.4, (LLLT): 9.3 ± 1.4		ELISA technique for gingival crevicular fluid (GCF) samples
Abdarazik et al., 2020 [32] Cairo, Egypt	- Fixed orthodontic appliances + 0.016 × 0.022-inch SS+ NiTi closed-coil springs (150 g) from the power arm mesial to U3 to the miniscrew for retraction U3.	TADs between 5 and 6	2nd,6th, 14th, 16th week, and at the end of canine retraction	CTM (mm): (statistically significant with p-value <0.01) Just immediately before starting of retraction: U: 12.000; p-value: 0.072 2nd week: U: 2.500; p-value: 0.001 6th week: U: 5.500; p-value: 0.006 16th week: U: 27.000; p-value: 0.955 at the end of canine retraction: U: 25.000; p-value: 0.779 TTM (weeks): (statistically significant with p-value <0.01)	Molar anchorage loss: (p-value = NR) (the method of assessing not reported), there was a statistically significant difference between FTMPF and LLLT groups; it was higher in the LLLT Periodontal probing depth: (p-value = NR) (The method of assessing not reported), there was no statistically significant difference between FTMPF and LLLT groups	Measurements were done using 3D scanned study models and 3D superimposition.
Türker et al., 2021 [36] Kayseri, Turkey	- Roth brackets+ 0.016 × 0.022-inch SS+ NiTi closed-coil springs (150 g) between miniscrews and slider hook for retraction U3.	TADs between 5 and 6	Every two weeks	RTM (mm/week): p-value = 0.124 Piezocision: 3.68 ± 0.42, LLLT: 3.89 ± 0.41	Canine and 1st molar angulation: Using lateral cephalometric analysis Canine tipping: p-value = 0.711 Piezocision: 2.92 ± 1.76, LLLT: 2.72 ± 1.52 1st molar: p-value = 0.886 Piezocision: –1.24 ± 1.72, LLLT: –1.16± 1.76	Measurements were done using 3D scanned study models and 3D superimposition.

**Table 4:** Protocols of the ongoing study registered at the WHO ICTRP NR: not reported; WHO ICTRP: World Health Organization International Clinical Trials Registry Platform Search Portal

Study ID	Trial name or title	Study design	Intervention + treatment comparison	Sample size/age/gender	Outcomes
CTRI/2018/05/014328	Comparison of micro osteoperforation and low-level laser therapy on the rate of retraction - an in vivo study.	NR	Low-level laser therapy versus micro- osteoperforation	30/18-48/both (male, females)	Primary outcomes: velocity of tooth movements; secondary outcomes: NR

**Table 5:** Additional characteristics of the protocols of ongoing studies. NR: not reported; U4: upper first premolar; U3: upper canine

Study ID	Setting	Orthodontic aspects	Technical aspects of interventions	Notes
CTRI/2018/05/014328	Department of orthodontics, Sri Hasanamba dental college and hospital, India	Baseline Characteristics: - Indication of U4s' extraction. - Age range: between 18 and 48 years. - Average skeletal pattern. - 5mm space at least is available for U3 retraction.	NR	This study is currently in phase 2; starting date: December 12, 2017; completion date: NR

Four completed RCTs [23,32-34] and two CCTs [35,36], including 154 patients, were included in this review. The age range was from 15 to 29 years. One study included only female patients [32], whereas, in another study, the number of females was less than that of males [35]. In three studies, the number of females was more than that of males [33,34,36]. Sex distribution was not given in only one study [23]. 

Four of the included studies were of a split-mouth design (SMD) [33-36], and two were of a compound (COMP) design (both parallel and split-mouth) [23,32]. In the compound-design studies, the surgical sides in the experimental groups were compared with the non-surgical sides in the other experimental groups since the contralateral sides in these groups did not undergo any acceleration (traditional orthodontic treatment only) [23,32]. The other four studies conducted this comparison directly without any non-accelerated control group [33-36].

Five studies compared surgical with physical interventions (i.e., low-level laser therapy {LLLT}), whereas the sixth study compared a surgical intervention with a pharmacological one (i.e., prostaglandin E1). The surgical interventions ranged from clearly invasive (traditional corticotomy [33-35], full-thickness mucoperiosteal flap FTMPF [32]), to minimally invasive interventions (micro-osteoperforations {MOPs} [23] and flapless piezocision [36]).

All the retrieved studies included patients who required canine retraction after premolar extraction [23,32-36]. All included patients underwent extraction-based treatments. The retraction was performed on canines after maxillary first premolars extraction. Extraction was performed at the beginning of treatment before initiating leveling and alignment in three studies [23,35,36] and after the leveling and alignment completion in the other three studies [32-34]. Follow-up assessment times varied from two weeks [34], three months [23,36], four months [33], and to the point of completion of canine retraction [32,35]. Measurement of tooth movement was expressed as the "rate of tooth movement" (RTM) in four studies [23,33,35,36], "cumulative tooth movement" (CTM) in one study, "time of tooth movement" (TTM) in two studies [32,35], and one study investigated the sRANKL concentration [34]. Five studies used temporary anchorage devices TADs [23,32-34,36], whereas the sixth study depended on a tip backbend for anchorage [35]. Concerning the method used to measure the speed of tooth movement, one of the included studies used a digital intra-oral caliper [23], one study used the ELISA technique for gingival crevicular fluid (GCF) samples [34], two studies evaluated plaster models using electric digital calipers [33,35], whereas two studied relied on 3D scanned study models for obtaining the measurements [32,36].

Risk of Bias of the Included Studies

**Figure 2:**
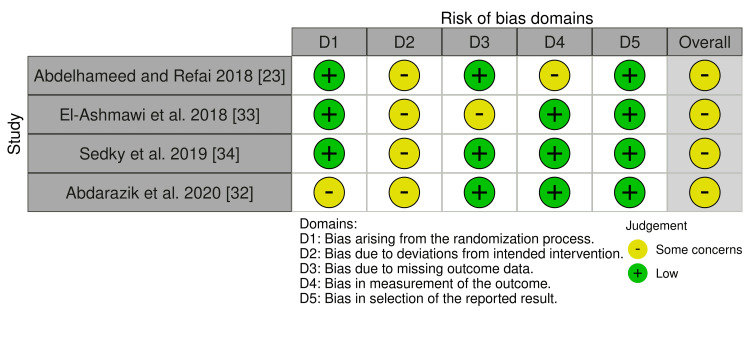
Risk of bias summary of RCTs. Low risk of bias (the plus sign); some concern of bias (the minus sign) RCTs: randomized controlled trials

**Figure 3:**
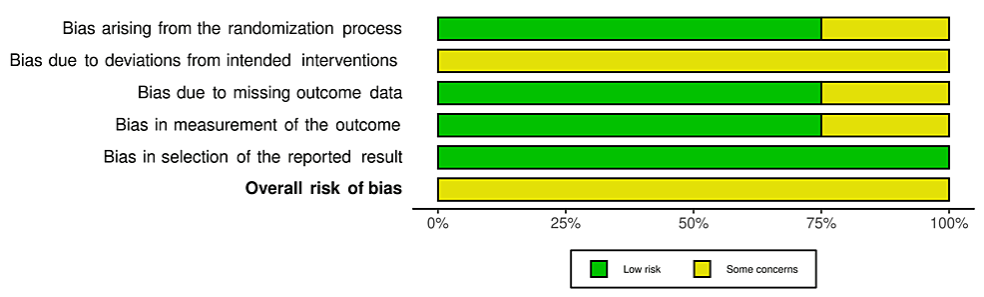
The overall risk of bias score for each field of RCTs. RCTs: randomized controlled trials The graph is based on Abdelhameed and Refai, 2018 [23], El-Ashmawi et al., 2018 [33], Sedky et al., 2019 [34], and Abdarazik et al., 2020 [32].

**Figure 4:**
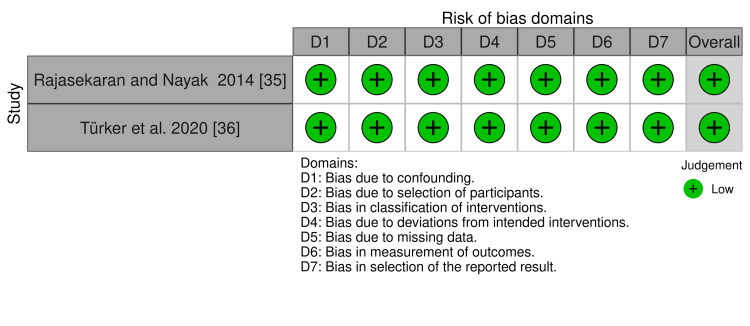
Risk of bias summary of CCTs. Low risk of bias (the plus sign). CCTs: controlled clinical trials

The risk of bias in the included RCTs is shown in Figure 2, and the overall risk of bias for each domain is presented in Figure 3. All RCTs were assessed as having "some concern of bias" [23,32-35]. "Some concern of bias" was the dominant feature among the RCTs. Bias due to deviations from intended interventions (effect of assignment to intervention; effect of adhering to intervention) was the most doubtful domain (i.e., "some concern" in 100% of the four studies). The risk of bias assessment of CCTs studies is shown in Figure 4. The studies were at "low risk of bias."

Effects of Interventions: Primary Outcomes

Surgical versus physical interventions: Five studies compared the different types of surgical interventions with low-level laser therapy (LLLT) in the acceleration of canine retraction [23,32-34]. El-Ashmawi et al. evaluated the effect of "traditional corticotomy" versus "LLLT" in a split-mouth RCT [33]. Regarding the rate of canine retraction, no statistically significant differences were found between the “corticotomy” and the “LLLT” sides at any assessment time (mean 0.23 mm, 95% CI: -0.7 to 1.2, p = 0.64).

Türker et al. estimated the effects of “piezocision” and “LLLT” on RTM in a split-mouth CCT [36]. In the first month, the rate of upper canine retraction was statistically greater on the “LLLT” side compared with the “piezocision” side (p = 0.002). However, in the second and third months of upper canine retraction, no statistically significant differences between the two sides were observed (p = 0.377, p = 0.667), respectively. When taking into account the overall assessment time, the effects of “LLLT” and “piezocision” on OTM were similar (p = 0.124), although the LLLT seemed to be more effective than the “piezocision” procedure during the first month.

Abdelhameed and Refai investigated the effect of “MOPs” versus “LLLT” in conjunction with “MOPs+LLLT” on the RTM in a compound-design RCT [23]. They found an increase in the rate of upper canine retraction in the accelerated sides (“MOPs” as well as “LLLT”) when compared with the non-accelerated sides, with statistically significant differences at all assessment times (p< 0.05). The rate of canine retraction was accelerated in the “MOPs” and the “LLLT” sides by 1.6-fold and 1.3-fold, respectively, when compared with the non-accelerated sides. Also, they demonstrated that the “MOPs” procedure was slightly more effective than the “LLLT” in the acceleration of upper canine retraction, though the difference was not statistically significant. The high heterogeneity, as well as the difference in the applied interventions between the previous studies, did not allow for quantitative synthesis of the data [23,33,36]. Abdarazik et al. estimated the effect of “full-thickness mucoperiosteal flap (FTMPF elevation only versus “LLLT” on “cumulative tooth movement” (CTM) and “time of tooth movement” (TTM) in a two-arm compound-design RCT [32]. Regarding the “time of tooth movement,” a significant decrease in the total canine retraction time was observed when comparing the accelerated sides with the non-accelerated ones. No statistically significant differences were found between “FTMPF” and “LLLT” in the overall study period regarding “cumulative tooth movement” (p = 0.728) and “time of tooth movement” (p = 0.298). Moreover, “FTMPF” and “LLLT” could achieve an acceleration of OTM by 25 and 20%, respectively.

Sedky et al. evaluated and compared the effect of “traditional corticotomy” versus “LLLT” on RANKL release during the OTM in a split-mouth-design RCT [34]. This study reported that both “corticotomy” and “LLLT” could increase the RANKL release during the OTM, leading to directly affecting bone remodeling and the rate of OTM. There was no statistically significant difference between the two sides on the third day and the 15th day after the intervention (p = 0.685 and p = 0.400, respectively). The difference in the times or the method of outcome evaluation was the reason to prevent including the two previous studies in the meta-analysis [32,34].

Surgical versus pharmacological interventions: Rajasekaran and Nayak evaluated the effect of corticotomy versus prostaglandin E1 injection on RTM and "time of tooth movement" (TTM) in a split-mouth-design CCT [35]. They demonstrated that the corticotomy improved the RTM better than prostaglandins with a statistically significant difference (p = 0.003), as the average RTM was 0.36 ±0.05 mm/week on the prostaglandin side, whereas it was 0.40 ±0.04 mm/week on the corticotomy side. There was also a difference in the time of tooth movement between the two interventions. The “time of tooth movement” in the corticotomy group (13 weeks) was less than compared with the prostaglandin group (15 weeks). For more details, a synopsis of quantitative conclusions for the primary outcome of each study is presented in Table 6. 

**Table 6:** A synopsis of quantitative conclusions for the primary outcome in each study RTM: rate of tooth movement; TTM: time of tooth movement; CTM: cumulative tooth movement; NAC: non-accelerated control; MOPs: micro-osteoperforations; LLLT: low-level laser therapy; CFO: corticotomy-facilitated orthodontics; FTMPF: full-thickness mucoperiosteal flap; NR: not reported

Study/setting	Primary outcome	Time points of measurement	Surgical group (mean ±SD)		Non-surgical group (mean ±SD)		p-Value
Rajasekaran and Nayak, 2014 [35] India	RTM (mm/week)	Mean of total RTM	Corticotomy 0.40 ± 0.04		prostaglandin E1 0.36 ± 0.05		0.003
	TTM (weeks)	Median of total weeks	13		15		NR
Abdelhameed and Refai, 2018 [23] Minya, Egypt	RTM (mm/week)	2 weeks	MOPs 1.3 ± 0.12	NAC 0.63 ± 0.62	LLLT 0.98 ±0.27	NAC 0.66 ± 0.55	<0.05
		4 weeks	2.16 ± 0.27	1.31 ± 0.23	1.81 ± 0.39	1.28 ± 0.48	<0.05
		6 weeks	2.92 ± 0.73	1.8 ± 0.66	2.38 ± 0.27	1.76 ± 0.83	<0.05
		8 weeks	3.43 ± 0.66	1.97 ± 0.76	2.63 ± 0.87	1.82 ± 0.63	<0.05
		10 weeks	3.92 ± 0.88	2.56 ± 0.83	3.26 ± 0.89	2.43 ± 0.23	<0.05
		12 weeks	4.33 ± 0.64	2.82 ± 0.39	3.72 ± 0.71	2.77 ± 0.37	<0.05
El-Ashmawi et al., 2018 [33] Cairo, Egypt	RTM (mm/week)	2 weeks	Corticotomy -0.95 ± 1.03		LLLT -0.97 ± 1.08		0.956
		3 weeks	-0.78 ± 0.62		-0.94 ± 0.91		0.525
		5 weeks	-1.76 ± 0.77		-2.02 ± 0.93		0.410
		7 weeks	-1.98 ± 1.14		-2.07 ± 1.07		0.812
		9 weeks	-2.80 ± 0.84		-2.85 ± 1.09		0.874
		11 weeks	-3.18 ± 1.21		-3.02 ± 1.18		0.607
		13 weeks	-3.81 ± 0.99		-3.77 ± 1.19		0.874
		15 weeks	-3.97 ± 1.40		-3.94 ± 1.27		0.945
		17 weeks	-4.32 ± 1.29		-4.55 ± 1.72		0.566
		Mean of total RTM	4.32 ±1.29		4.55 ± 1.7		>0.05
Sedky et al., 2019 [34] Cairo, Egypt	sRANKL concentration (pg\ml)	1 day before the intervention	CFO 3.8 ± 1,4		LLLT 3.8 ± 1.4		NR
		3rd day after intervention	10.2 ± 1.2		10.0 ± 1.9		0.685
		15th day after intervention	8.9 ± 1.4		9.3 ± 1.4		0.400
Abdarazik et al., 2020 [32] Cairo, Egypt	CTM (mm)	The median of total CTM	FTMPF 6.77		LLLT 6.88		NR
	TTM (weeks)	The median total weeks	17.14		17.87		NR
Türker et al., 2020 [36] Kayseri, Turkey	RTM (mm/week)	4 weeks	Piezocision 1.34 ± 0.16		LLLT 1.52 ± 0.18		0.002
		8 weeks	1.21 ± 0.14		1.25 ± 0.17		0.377
		12 weeks	1.18 ± 0.16		1.16 ± 0.13		0.667
		The mean of total RTM	3.68 ± 0.42		3.89 ± 0.41		0.124

Effects of Interventions: Secondary Outcomes

Four studies evaluated secondary outcomes [32,33,35,36]. Molar anchorage loss was assessed in three studies [32,33,35]. Rajasekaran and Nayak found no statistically significant difference between the corticotomy and prostaglandin groups (p = 0.67) [35]. El-Ashmawi et al. found no statistically significant difference between corticotomy and LLLT sides at any assessment time (MD 0.33 mm, CI 95%: -1.22- 0.55, p = 0.45) [33]. Conversely, Abdarazik et al. reported a statistically significant difference between the FTMPF and LLLT groups; it was greater in the LLLT group [32].

Pain and swelling were evaluated in two included trials [33,35]. According to Rajasekaran and Nayak, the patients reported mild swelling and pain during the first week on the side of the corticotomy [35]. On the side of prostaglandin, all patients suffered from acute pain at times of injections. The intensity was high and persisted up to three days from the time of injection in most of the patients. However, El-Ashmawi et al. [33] reported that 70% of patients complained of swelling on the corticotomy side, while 10% of total patients suffered from the swelling on both corticotomy and LLLT sides. Eighty-five percent of patients reported post-surgery pain. It was more severe on the corticotomy side.

The crestal bone height changes and root length were assessed by Rajasekaran and Nayak, who observed no statistically significant difference between the corticotomy and prostaglandin groups (p = 0.08) [35]. Periodontal probing depth was evaluated in one study only, and no statistically significant difference between FTMPF and LLLT was found [32].

The changes in canine and first molar angulations were tested by Türker et al., and no statistically significant differences were found in the canine and first molar angulations between the piezocision and LLLT sides during the three-month observation period [36].

The Strength of the Evidence According to the GRADE Guidelines

Based on the GRADE recommendations, the strength of evidence for the rate of orthodontics tooth movement and the adverse effects ranged from "very low" to "low" (Table 7). The decline in the strength of the evidence occurred because of the risk of bias [23,32,33,35,36], indirectness [23,32], and imprecision [23,32,33,35,36].

**Table 7:** Summary of the findings according to the GRADE guidelines for the included trials ^a, g^Decline one level for risk of bias (bias due to deviations from intended interventions, large losses to follow-up), and one level for imprecision* [33]. ^b^Decline one level for risk of bias (nonrandomized trial), and one level for imprecision* [36]. ^c, f, i, j^Decline one level for risk of bias (nonrandomized trial), and one level for imprecision* [35]. ^d^Decline one level for risk of bias (bias due to deviations from intended interventions), one level for indirectness**, and one level for imprecision* [23]. ^e, h, k^Decline one level for risk of bias (bias arising from the randomization process, bias due to deviations from intended interventions), one level for indirectness**, and one level for imprecision* [32]. *Limited number of trials. **Outcome is not directly related. CI: confidence interval; SMD: split-mouth design; COMP: compound design; MD: mean difference; LLLT: low-level laser therapy; FTMPF: full-thickness mucoperiosteal flap

Quality assessment criteria						Summary of findings				Comments
No. of studies	Risk of bias	Inconsistency	Indirectness	Imprecision	Other considerations	No. of patients	Effects		Certainty	
							Absolute (95% CI)	Relative (95% CI)		
Rate of upper canine retraction accelerated by surgical and non-surgical interventions										
1 RCT (SMD)	Serious	Not serious	Not serious	Serious	None	20	-	Mean 0.23 mm, 95% CI, -0.7 to 1.2	Low ⊕⊕⊖⊖^a^	The difference between corticotomy and LLLT was not statistically significant (p = 0.64)
1 CCT (SMD)	Serious	Not serious	Not serious	Serious	None	20	-	Not estimable	Low ⊕⊕⊖⊖^b^	The difference between piezocision and LLLT was not statistically significant (p = 0.124)
1 CCT (SMD)	Serious	Not serious	Not serious	Serious	None	32	-	Not estimable	Low ⊕⊕⊖⊖^c^	The difference between corticotomy and prostaglandin was statistically significant (p = 0.003)
1 RCT (COMP)	Serious	Not serious	Serious	Serious	None	30	-	Not estimable	Very low ⊕⊖⊖⊖^d^	MOPs intervention was more efficient than the application of LLLT. The high heterogeneity, and the difference in the applied interventions, as well as the difference in the times or the method of outcome evaluation, could not allow for conducting quantitative synthesis of the previous findings.
Time need for upper canine retraction accelerated by surgical and non-surgical interventions										
1 RCT (COMP)	Serious	Not serious	Serious	Serious	None	32	-	Not estimable	Very low ⊕⊖⊖⊖^e^	The difference between FTMPF and LLLT was not statistically significant (p = 0.298)
Adverse effects: anchorage loss										
1 CCT (SMD)	Serious	Not serious	Not serious	Serious	None	32	-	Not estimable	Low ⊕⊕⊖⊖^f^	The difference between corticotomy and prostaglandin was not statistically significant (p = 0.67)
1 RCT (SMD)	Serious	Not serious	Not serious	Serious	None	20	-	MD 0.33mm, 95% CI, -1.22 - 0.55	Low ⊕⊕⊖⊖^g^	The difference between corticotomy and LLLT was not statistically significant (p = 0.45)
1 RCT (COMP)	Serious	Not serious	Serious	Serious	None	32	-	Not estimable	Very low ⊕⊖⊖⊖^h^	The difference between FTMPF and LLLT was statistically significant. We could not pool the results of the 3 previous trials which evaluated this outcome to quantitative synthesis due to too high statistical heterogeneity
Adverse effects: the crestal bone height changes										
1 CCT (SMD)	Serious	Not serious	Not serious	Serious	None	32	-	Not estimable	Low ⊕⊕⊖⊖^i^	The difference between Corticotomy and prostaglandin was not statistically significant (p = 0.08)
Adverse effects: root length										
1 CCT (SMD)	Serious	Not serious	Not serious	Serious	None	32	-	Not estimable	Low ⊕⊕⊖⊖^j^	The difference between corticotomy and prostaglandin was not statistically significant (p = 0.08)
Adverse effects: periodontal probing depth										
1 RCT (COMP)	Serious	Not serious	Serious	Serious	None	32	-	Not estimable	Very low ⊕⊖⊖⊖^k^	There was no statistically significant difference in the average periodontal probing depth between the FTMPF group and the LLLT group.

Discussion

A significant increase can be seen in the number of studies dealing with the acceleration of orthodontic movement with various acceleration techniques. Although surgical acceleration methods have been extensively investigated, non-surgical methods also have had their share of a wide body of research. Information and evidence regarding the superiority of one acceleration method over another remain ambiguous.

According to this SR, there is no agreement between the studies regarding the dominance of surgical or non-surgical methods in accelerating OTM. Abdelhameed and Refai and Rajasekaran and Nayak found that surgical interventions were more effective than the non-surgical ones in OTM [23,35]. On the contrary, Türker et al. found that the non-surgical intervention was more effective than the surgical one in the first month of upper canine retraction [36]. However, considering the overall trial period, they found that the effect of the surgical and non-surgical intervention on OTM was similar. Moreover, Abdarazik et al., El-Ashmawi et al., and Sedky et al. mentioned that there was no difference between the surgical and non-surgical interventions regarding the acceleration of OTM [32-34].

Several factors may explain this inconsistency in results. Firstly, the difference in the characteristics of acceleration techniques used. Rajasekaran and Nayak [35] applied surgical intervention (corticotomy) versus biological one (prostaglandin), while the other studies compared surgical interventions and physical ones all represented by LLLT [23,32-34,36].

However, acceleration methods affect the OTM through different mechanisms. Prostaglandins, which are one of the pharmacological agents, influence at the cellular level where they increase directly osteoclasts' number leading to stimulating bone resorption [8]. While LLLT, which falls within the physical methods, affects the cell at the molecular and chemical levels that stimulates the production of adenosine triphosphate (ATP) and decreases the levels of reactive oxygen species (ROS) [37]. This structural effect stimulates the proliferation and differentiation of various cells, such as osteoblasts and osteoclasts, leading to OTM acceleration [38]. On the other hand, the surgical methods depend on the regional acceleratory phenomenon RAP, which can be described as a decrease in cortical bone density due to increased osteoclastic activity following surgical wounding of the cortical bone [39]. This difference in the mechanisms affecting osteoclasts may be specifically one of the reasons for the difference in the findings among studies.

Secondly, there was a difference in the application protocols between techniques. Abdelhameed and Refai, in their study, repeated the application of MOPs every two weeks (i.e., six times over three months), while the surgical intervention in the other studies was applied only once [23,32-36]. Repeated application of the surgical technique implies continuous activation of regional acceleratory phenomenon (RAP), which begins within a few days of surgery, and typically peaks at one to two months, then decline over time [40]. This could explain why the surgically induced acceleration in Abdelhameed and Refai trial was superior to that of non-surgical methods and was not similar to findings of surgical interventions in the other studies [32-36].

When LLLT is considered a choice for acceleration, using an appropriate protocol is essential. In the present SR of LLLT studies, the wavelength of the laser beam was similar among them except for one study, which used an 810 ± 10 nm wavelength [23,32-34,36]. However, the type of the employed laser, energy density, the frequency of laser irradiation (number of applications), and the total irradiation time per tooth were not similar among these studies. Despite the inconsistent protocols, they concluded that the LLLT effectively accelerated the OTM.

When considering the factors affecting the rate of OTM, gender and age should be taken into account. Hormonal changes that occur monthly in women are associated with fluctuations in bone turnover [41]. On the other hand, young patients show a greater OTM rate than older adults [42]. Unfortunately, in this review, the effect of gender and age was not isolated because of the lack of data provided by the included studies.

In extraction‑based treatments, the timing of extracting premolars may play an important role in affecting the OTM. The inflammatory response following the extraction and evoking a RAP that occurs in the healing process of the alveolar sockets [43] can increase the rate of OTM. However, The velocity of OTM into an extraction site remains dialectical [44]. All the studies in the present SR enrolled patients who required upper first premolar extraction, but there was no agreement on the extraction timing [23,32-36]. Extraction was performed before initiating leveling and alignment in three studies [23,35,36], whereas it was done in the other studies after the leveling and alignment completion [32-34].

Concerning the side effects, three included trials investigated the unwanted tooth movements (molar anchorage loss) associated with surgical and non-surgical acceleration methods. No significant molar anchorage loss was reported in two studies [33,35]. Conversely, the third study found a statistically significant loss of molar anchorage in the non-surgical intervention side (i.e., the LLLT side) compared with the surgical intervention side (FTMPF) [32]. This may be explained because the surgical technique used in this study relied solely on a full-thickness mucoperiosteal flap elevation without any cortical bone injury; therefore, the effect of regional acceleration was minimal and did not extend back to the posterior segments. On the other hand, LLLT was applied on an entire region rather than a specific point so that the irradiation may have extended to the tissues surrounding the first molar. The results of the three previous trials could not be pooled in a quantitative synthesis due to high statistical heterogeneity. Concerning the crestal bone height changes, root length, and periodontal probing depth, the differences between the surgical and non-surgical were not statistically significant.

Limitations of the current review

Only four RCTs and two CCTs were found and included in this SR. The strength of evidence ranged from “low” to “very low.” Therefore, there is a need for high-quality, well-designed RCTs with an additional number of patients to arrive at stronger conclusions.

Upper first-premolars extraction‑based treatments followed by canine retraction were conducted in all groups of the included trials. No trials were found in the medical literature comparing surgical and non-surgical methods in the acceleration of teeth decrowding, incisors' retraction, en-masse retraction, molar intrusion, and other orthodontic tooth movement strategies.

The high heterogeneity among the retrieved studies, the difference in the applied interventions, and the variability in the assessment times or the outcome evaluation methods did not allow for quantitative synthesis of the findings. Statistical heterogeneity was also evident between the trials that evaluated the side effects of surgical and non-surgical acceleration techniques. Therefore, a meta-analysis of the related results was not possible.

## Conclusions

There is "very low" to "low" evidence that the surgical interventions and the non-surgical ones have a similar accelerating effect on upper canine retraction. There is "very low" to "low" evidence that the surgical interventions are superior to the non-surgical ones. There is "low" to "very low" evidence that the reported side effects associated with surgical and non-surgical methods are similar.

As the quality of evidence of the reported results ranged from "low" to "very low." Therefore, we confirm the need for more well‑conducted RCTs to compare surgical and non-surgical interventions regarding the acceleration of orthodontic tooth movement in different cases of malocclusion. Future studies should take into account the patient-reported outcomes and side effects accompanied by both surgical and non-surgical acceleration procedures.

## Appendices

**Table 8:** Excluded studies and the reasons beyond exclusion

Study	Reason for exclusion
Showkatbakhsh R, Jamilian A, Showkatbakhsh M. The effect of pulsed electromagnetic fields on the acceleration of tooth movement. World J Orthod. 2010, 11(4): 52-6.	The study did not compare surgical with non-surgical acceleration methods, and it evaluated the pulsed electromagnetic field group vs. non-accelerated control group
El Ashmawi NM, Selim EM, Fayed MS, El-Beialy AR. Comparison between the effect of corticotomy and low level laser therapy on periodontal health. Egypt Dent J. 2016, 62:1113-9.‏	The study did not evaluate the effect of corticotomy and low-level laser therapy on tooth movement acceleration, it compared the effect of the two methods on periodontal health.
Farid KA, Eid AA, Kaddah MA, Elsharaby FA. The effect of combined corticotomy and low level laser therapy on the rate of orthodontic tooth movement: split mouth randomized clinical trial. Laser Therapy. 2019, 28(4):275-83.‏	The study did not separately apply surgical and non-surgical acceleration methods, but they were applied together in the same group of patients
Jumayev M. Comparing the Effects of Piezocision and Low Level Laser Therapy in Orthodontic Canine Distalization. Health Sciences Institute: Turkey; 2019.	A thesis: The study did not separately apply surgical and non-surgical acceleration methods, but they were applied together in the same group of patients
Yousif AA, Abo-Taha NF, Essa EF. Accelerated canine retraction by corticotomy assisted or periodontal distraction. Egypt Dent J. 2019, 65:3221-31.	The study did not separately apply surgical and non-surgical acceleration methods, but they were applied together in the same group of patients
ACTRN12615000593538: The effects of micro-osteoperforations on orthodontic root resorption and tooth movement.	Ongoing trial (protocol): the study is not comparing surgical versus non-surgical acceleration methods. The control group is non-accelerated, whereas the experimental group includes the micro-osteoperforations acceleration method.
ChiCTR1900024297: Accelerate orthodontic tooth movement by piezocision assisted orthodontics: a randomized controlled trial.	Ongoing trial (protocol): the study is not comparing surgical versus non-surgical acceleration methods. The control group is non-accelerated, whereas the experimental group includes the piezocision acceleration method.
CTRI/2018/04/013181: Effect of a submucosal injection of Platelet Rich Plasma on the rate of orthodontic tooth movement - A split mouth, randomised controlled trial - PRP.	Ongoing trial (protocol): the study is not comparing surgical versus non-surgical acceleration methods. The control group is non-accelerated, whereas the experimental group includes the platelet rich plasma acceleration method.
CTRI/2018/07/015109: Effectiveness of combined piezocision and low-level laser therapy in reducing orthodontic treatment duration and patient discomfort â?? A randomized controlled trial.	Ongoing trial (protocol): the study is not separately applying surgical and non-surgical acceleration methods, but they are applied together in the same group of patients.
CTRI/2020/04/024453: Effectiveness of piezocision assisted corticotomy and low-level laser therapy in enhancing rapid maxillary canine retraction: a randomised controlled trial.	Ongoing trial (protocol): the study is not separately applying surgical and non-surgical acceleration methods, but they are applied together in the same group of patients.
